# Chronic Alcohol Intoxication and Cortical Ischemia: Study of Their Comorbidity and the Protective Effects of Minocycline

**DOI:** 10.1155/2016/1341453

**Published:** 2016-06-22

**Authors:** Enéas Andrade Fontes-Júnior, Cristiane Socorro Ferraz Maia, Luanna Melo Pereira Fernandes, Walace Gomes-Leal, Allan Costa-Malaquias, Rafael Rodrigues Lima, Rui Daniel Prediger, Maria Elena Crespo-López

**Affiliations:** ^1^Laboratory of Molecular Pharmacology, Institute of Biological Sciences, Federal University of Pará, 66075-110 Belém, PA, Brazil; ^2^Laboratory of Pharmacology of Inflammation and Behavior, Institute of Health Sciences, Federal University of Pará, 66075-110 Belém, PA, Brazil; ^3^Laboratory of Experimental Neuroprotection and Neurodegeneration, Institute of Biological Sciences, Federal University of Pará, 66075-110 Belém, PA, Brazil; ^4^Laboratory of Functional and Structural Biology, Institute of Biological Sciences, Federal University of Pará, 66075-110 Belém, PA, Brazil; ^5^Department of Pharmacology, Center of Biological Sciences, Federal University of Santa Catarina, 88040-900 Florianópolis, SC, Brazil

## Abstract

Chronic alcohol intoxication (CAI) increases both morbidity and mortality of stroke patients. Despite the high prevalence of CAI and ischemic stroke, studies addressing their comorbidity and/or protective alternatives remain scarce. Thus, the influence of CAI on both stroke outcome and minocycline treatment (recognized for its neuroprotective effect) was investigated. Female Wistar rats (35 days old) were treated with water or ethanol (6.5 g/kg/day, 22.5% w/v) for 55 days. Then, focal ischemia was induced by endothelin-1 in the motor cortex. Two hours later, four doses of 50 mg/kg of minocycline every 12 hours followed by five doses of 25 mg/kg every 24 hours were administered. Behavioral performance (open field and rotarod tests) and immunohistochemical (cellular density, neuronal death, and astrocytic activation) and biochemical (lipid peroxidation and nitrite levels) analyses were performed. CAI increased motor disruption, nitrite and lipid peroxidation levels, and neuronal loss caused by ischemia, whereas it reduced the astrogliosis. Minocycline was effective in preventing the motor and tissue damage caused by stroke. However, these effects were attenuated when CAI preceded stroke. Our data suggest that CAI beginning in adolescence contributes to a worse outcome in ischemic stroke survivors and reduces the benefits of minocycline, possibly requiring adjustments in therapy.

## 1. Introduction

Heavy alcohol drinking significantly increases both morbidity and mortality of stroke patients [1]. It is also a major risk factor for cerebrovascular diseases [1]. Female and young adults have been highlighted as major groups with the highest growth in alcohol consumption for the next decades [2]. In Brazil, female population was already pointed as the group with the largest increase in ethanol consumption during the period 2006–2012 [3]. Chronic alcohol intoxication (CAI) usually begins during adolescence triggering neuroinflammatory and oxidative processes, leading to significant neuronal loss (these mechanisms are also shared by ischemic stroke) [4]. Despite the high prevalence of CAI and ischemic stroke found in clinical practice, few preclinical and clinical studies have addressed the consequences of their comorbidity.

Our preliminary results revealed motor deficits and increased microglial activation in ischemic rats previously intoxicated with ethanol [5], leading us to ask about the role of oxidative stress in the comorbidity. Moreover, treatment with minocycline was able to partially prevent these alterations. Recent studies proposed minocycline as a promising therapy for stroke. Clinical improvement of the neurological outcomes of the patients was observed after 30 days of treatment with this drug [6]. However, to date no study has showed the possible role of oxidative stress in the alterations of such response in the comorbidity with CAI.

Minocycline is a potent anti-inflammatory/antibiotic tetracycline with well-established neuroprotective effects [7, 8]. In the present study, we investigated the putative neuroprotective effects of minocycline treatment in ischemic rats chronically intoxicated with ethanol since adolescence, with a special focus on the analysis of glia activation and oxidative stress.

## 2. Materials and Methods

### 2.1. Animals and Ethical Aspects

Sixty female Wistar rats (35 days old) were housed (21 ± 2°C; 12 h light/dark cycle) with food and water* ad libitum*. All efforts were carried out to reduce the number of animals and minimize their suffering. This study followed the NIH Guide for the Care and Use of Laboratory Animals and it was approved by the Committee for Ethics in Experimental Research with Animals of the Federal University of Pará (license number BIO007-09).

### 2.2. Study Design

**Figure 1:**
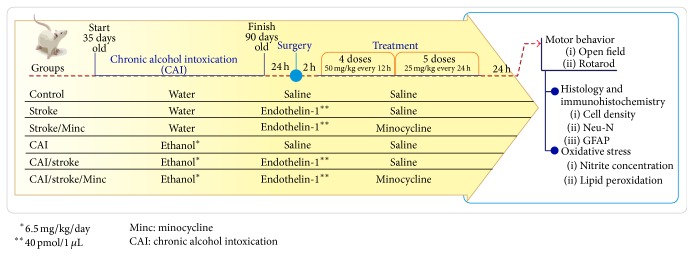
Experimental design.

Animals were randomly divided into six groups (10 animals/group) (Figure 1). They received orally (gavage) distilled water or ethanol (6.5 g/kg/day, 22.5% w/v) once a day, for 55 days (animals were 90 days old at the end of this treatment). Then, focal ischemia was induced by stereotaxic microinjection of 1 *μ*L (40 pmol) endothelin-1 (ET-1), a potent vasoconstrictor, into the left motor cortex (2.3 mm lateral and 1.2 mm posterior from bregma and 0.4 mm below the pial surface).

Two hours after ischemia induction (counted after the removal of the cannula), animals received saline or minocycline intraperitoneally (four doses of 50 mg/kg every 12 hours followed by five doses of 25 mg/kg every 24 hours). The latter treatment was according to that previously described [11].

No death was observed within the 55 days of the ethanol treatment as well as after the treatments with ET-1 and/or minocycline (data not shown). All the behavioral, histopathological, immunohistochemical, and biochemical analyses were performed by an experienced experimenter who was unaware of the experimental group of the animals tested.

### 2.3. Behavioral Assays

Twenty-four hours after the last drug administration, animals were acclimated for 1 h in a room with attenuation of noise levels and low illumination (12 lux). Then, the open field (5 min) and the rotarod (three successive trials of 3 min each one, on the rotating rod at 15 rpm) tests were carried out [5].

### 2.4. Biochemical Analysis

After behavioral assays, five animals per group were sacrificed by cervical dislocation and cerebral cortex was collected and processed for spectrophotometric analysis of lipid peroxidation (LPO, using malondialdehyde (MDA) as an indicator) and nitrite levels (an indirect marker of nitric oxide production), as previously described [12, 13]. Data were corrected according to the protein concentration of each sample [14]. Then, results were expressed as percentages of control groups.

### 2.5. Histological and Immunohistochemical Evaluations

Five animals per group (different from those for biochemical analysis) were deeply anesthetized (ketamine) and transcardially perfused with a solution of 4% paraformaldehyde diluted in 0.2 M phosphate buffer. Frozen sections of postfixed brains were stained with cresyl violet and they were analyzed as previously described [5, 15].

Coronal sections of cerebral cortex were also analyzed for Neu-N-positive neurons and GFAP-positive astrocytes by immunohistochemistry (1 : 500 and 1 : 1000, resp.). Briefly, slide-mounted sections were kept in an oven at 37°C for 30 min and rinsed once in 0.1 M phosphate buffer saline (PBS) for 5 min. To improve labeling intensity, sections were then pretreated in 0.2 M boric acid (pH 9.0) previously heated to 65°C for 25 min. Sections were further allowed to cool for about 20 min and incubated under constant agitation in 1% hydrogen peroxide solution in methanol for 20 min. Sections were then rinsed in 0.05% PBS/Tween (Sigma, USA) solution for 3 min (three times) and incubated with 10% normal horse (for anti-Neu-N antibody) or normal goat (for anti-GFAP antibody) serum in PBS for 30 min. Sections were then incubated with the primary antibody diluted in PBS for 2 h, rinsed in PBS/Tween solution for 3 min (3 times), and incubated with the biotinylated horse anti-mouse (anti-Neu-N antibody) or goat anti-rabbit (anti-GFAP antibody) secondary antibodies (Vector Laboratories, USA) diluted at 1 : 100 and 1 : 200, respectively, in PBS for 1 h. Sections were washed three times and incubated in the complex avidin-biotin-peroxidase (ABC Kit, Vector Laboratories, USA) for 45 min. Sections were then rinsed four times and DAB-reacted according to the protocol described elsewhere [5, 15]. After DAB reaction, sections were rinsed three times in 0.1 M phosphate buffer, dehydrated using alcohols and xylene, and covered with a coverslip.

**Figure 2:**
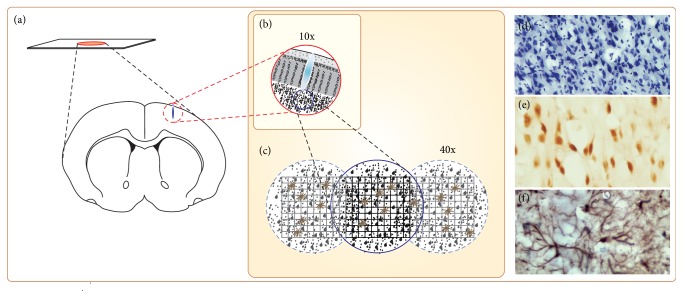
Method for histological and immunohistochemical evaluations in motor cortex. (a) Schematic representation of the brain slices; (b) 10x magnification; (c) 40x magnification; (d) cresyl violet-stained cells; (e) Neu-N positive cells; (f) GFAP-positive cells.

For quantitative assessments, the number of Neu-N-positive neurons and GFAP-positive astrocytes was evaluated by using a square 0.25 mm wide grid in the eyepiece of the microscope (Figure 2). This grid corresponds to an area of 0.0625 mm^2^. At least, three fields in the motor cortex per section and three sections per animal of each group were analyzed.

### 2.6. Statistical Analysis

All values were expressed as means ± SEM. Gaussian distribution was analyzed by Kolmogorov-Smirnoff test. Two-way analysis of variance (ANOVA) followed by* post hoc* Bonferroni's test was applied. Level of significance was set at *P* < 0.05.

## 3. Results

**Figure 3:**
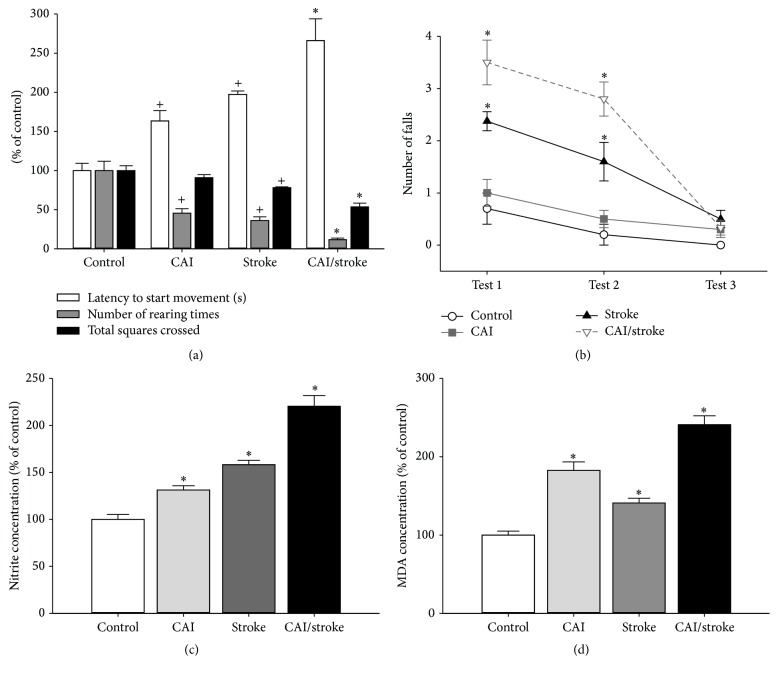
Chronic alcohol intoxication (CAI) worsened the ischemia (stroke) motor outcome and oxidative stress in motor cortex. (a) Open field; (b) rotarod (three sessions at 15 rpm); (c) nitrite concentration; (d) lipid peroxidation (malonaldehyde (MDA) concentration). Data: mean ± SEM [*n* = 10 (a, b), *n* = 5 (c, d)]. ^*∗*^ *P* < 0.05 versus all groups; ^+^ *P* < 0.05 versus control.

Chronic alcoholic intoxication (CAI) exacerbated the motor deficits induced by motor cortex ischemia in rats. CAI/stroke group showed high latency to initiate movements, low number of rearing times, and reduced distance travelled in the open field (Figure 3(a)). Also, an increase in the number of falls in rotarod test was detected for this group (Figure 3(b)).

Both CAI and ischemia increased* per se* the levels of both nitrite (Figure 3(c)) and LPO (Figure 3(d)) in cerebral cortex. A synergistic response was observed in the CAI/stroke group (Figures 3(c) and 3(d)).

**Figure 4:**
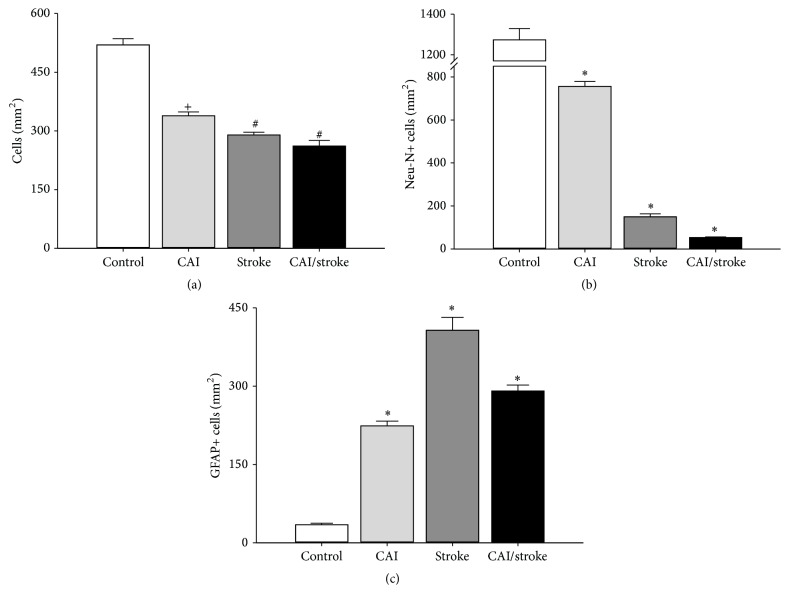
Chronic alcohol intoxication (CAI) increases neuronal loss and astrocytic activation caused by ischemia (stroke). (a) Total number of cells; (b) Neu-N-positive cells; (c) GFAP-positive cells. Data: mean ± SEM (*n* = 5). ^*∗*^ *P* < 0.05 versus all groups; ^+^ *P* < 0.05 versus control; ^#^ *P* < 0.05 versus control and CAI.

CAI did not significantly worsen the decrease in the number of cells caused by ischemia (Figure 4(a)), but it diminished the number of neuronal (Neu-N-positive) cells (Figure 4(b)) in the motor cortex. Both CAI and ischemia individually enhanced astrocytic (GFAP-positive) activation (Figure 4(c)). Interestingly, CAI attenuated the ischemia-induced increase of astrocytic activation (Figure 4(c)).

**Figure 5:**
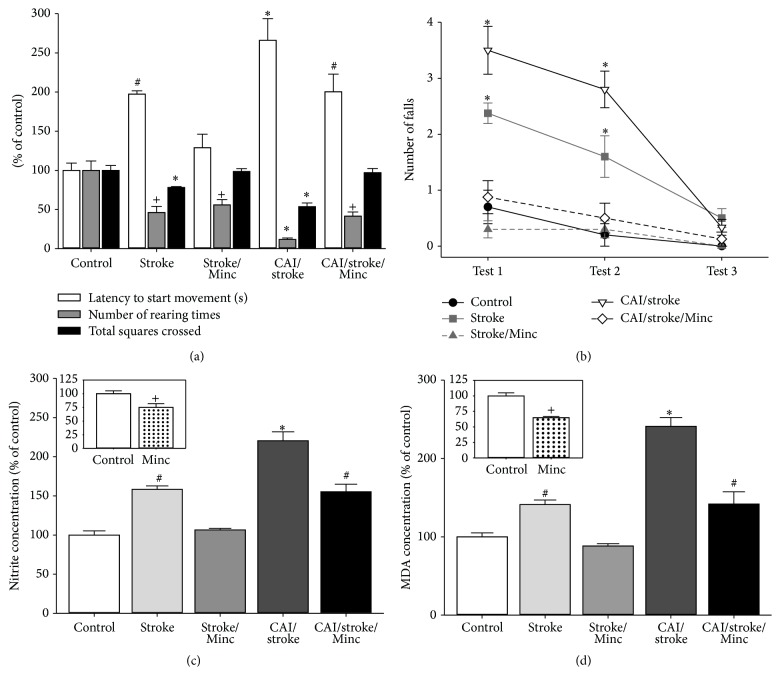
Chronic alcohol intoxication (CAI) reduces minocycline- (Minc-) induced protection in behavioral alterations and oxidative stress caused by ischemia (stroke). (a) Open field; (b) rotarod (three sessions at 15 rpm); (c) nitrite concentration; (d) lipid peroxidation (malonaldehyde (MDA) concentration). Data: mean ± SEM [*n* = 10 (a, b), *n* = 5 (c, d)]. ^*∗*^ *P* < 0.05 versus all groups; ^+^ *P* < 0.05 versus control; ^#^ *P* < 0.05 versus control and stroke/Minc.

Treatment with minocycline counteracted the motor impairment induced by CAI, ischemia, and their association in open field (Figure 5(a)) and rotarod (Figure 5(b)). Although minocycline was effective and it prevented oxidative stress caused by ischemia, it was only partially effective for reducing nitrite and MDA levels in the ischemic rats previously intoxicated with ethanol (Figures 5(c) and 5(d)).

**Figure 6:**
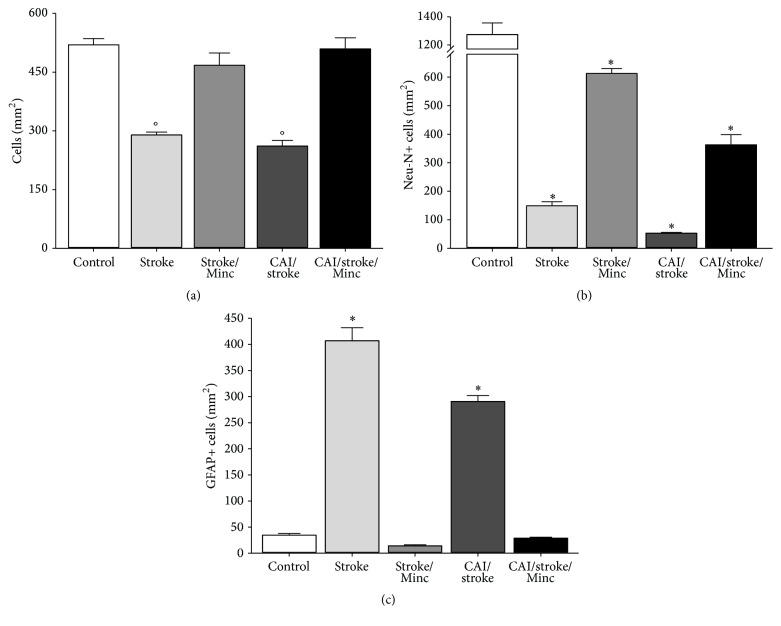
Chronic alcohol intoxication (CAI) limited beneficial effects of minocycline (Min) on neuronal loss and astrocytic activation caused by ischemia (stroke). (a) Total number of cells; (b) Neu-N-positive cells; (c) GFAP-positive cells. Data: mean ± SEM (*n* = 5). ^*∗*^ *P* < 0.05 versus all groups; °*P* < 0.05 versus stroke, stroke/Minc, and CAI/stroke/Minc.

Finally, CAI did not modify the protective effects of minocycline and it prevented both the ischemia-induced cellular death (Figure 6(a)) and astrocytic activation (Figure 6(c)). Nonetheless, the previous chronic ethanol intoxication reduced significantly the protective effects of minocycline against the neuronal loss caused by ischemia (Figure 6(b)).

## 4. Discussion

The heavy ethanol consumption has been epidemiologically highlighted as an independent risk factor for the prevalence and mortality of stroke patients [16]. However, preclinical studies about their comorbidity and/or its consequences for the therapy efficacy are scarce.

Our model of CAI (heavy and regular ethanol intake from adolescence to adulthood) reproduces the largest increase in ethanol consumption of human during a critical period of neurodevelopment [17]. Also, we used females because the last data in Brazil during the period 2006–2012 have pointed to female group as the group with the largest increase in ethanol consumption [3].

**Figure 7:**
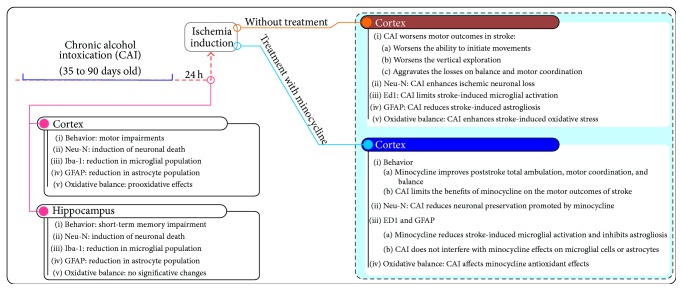
Overview of results already observed with this experimental design. Data from this paper and from previously described studies of our group [5, 9, 10].

Our previous results showed that the same CAI protocol leads to motor and memory impairments in rats, accompanied by a marked neuronal death and reduced microglial and astrocytic densities in both hippocampus and motor cortex [9, 10]. These deleterious consequences were more prominent in cerebral cortex with a major role for the increased oxidative stress (Figure 7).

Motor cortex is one of the main brain areas responsible for the clinical manifestations in stroke outcome (up to 42% of stroke survivors showed motor impairments with alterations of voluntary movements, closely associated with cortical function) [18]. Our results may indicate that ethanol intoxication would already induce significant motor changes as a starting point for a possible synergism in the comorbidity with stroke. Our preliminary results with the current model [5] highlighted this hypothesis revealing the development of motor incoordination and locomotor activity deficits associated with intense microglial activation in ischemic rats previously intoxicated with ethanol. Thus, this study advanced the investigation of CAI-ischemia interactions in three different levels (behavioral, cellular, and biochemical) with a special focus on the analysis of oxidative stress.

Focal ischemia in the motor cortex region decreased the spontaneous locomotor activity of rats in the open field and induced intense impairments of coordination, balance, and motor learning in the rotarod. These events were observed seven days after the ischemic induction, so they probably result due to the expansion of the ischemic core and penumbra areas after stroke, damaging the connection of cortex with striatum and cerebellum [19].

The current findings corroborate earlier results suggesting motor disruption in animals with endothelin-1-induced focal ischemia [20]. These results also agree with the clinical occurrence of most frequent manifestations in stroke survivors [21].

These behavioral alterations were accompanied by the exacerbated oxidative stress process (as revealed by high MDA and nitrite levels, with increases of about 40% and 60%, resp.) in cerebral cortex. In ischemic injury, oxidative stress is mainly triggered by glutamatergic excitotoxicity as a result of the activation of numerous calcium-dependent enzymes (especially neuronal nitric oxide synthase (nNOS)) that produce excessive amounts of oxygen and other reactive species. Later, inducible NOS (iNOS) contributes to high production of nitric oxide that plays an important role in neurodegeneration of the ischemic penumbra area. Also, increased production of superoxide radicals, by the action of xanthine oxidase (OX) and NADPH oxidase (NOX), takes place, primarily in glial cells and leukocytes [22].

In a similar way to that observed for focal ischemia, CAI also disrupted motor performance with a significant exacerbation of oxidative stress (increases of about 80% and 30% for MDA and nitrite levels, resp.). These results reinforce the involvement of ethanol in the production of reactive oxygen (O_2_^•^ and OH^•^, among others) and nitrogen (especially nitric oxide) species, peroxidation and fragmentation of macromolecules, mitochondrial damage, and neurodegeneration [23]. Ethanol also increases the activity of NOX, a protein complex responsible for the “respiratory burst” in phagocytic cells, generating large amounts of reactive species [24]. Other mechanisms involved in this process are the increase of phospholipase A2 activity (leading to oxidation, peroxidation, and epoxidation of fatty acids), oxidation of CYP2E1, deficits in removal mechanisms of reactive species, and depletion of endogenous antioxidants [25, 26].

Because the behavioral and neurochemical assessments were performed eight days after the last ethanol administration, a possible influence of a withdrawal syndrome cannot be ruled out. The long-term inhibitory effects of ethanol on glutamatergic neurotransmission can lead to adaptive changes including the upregulation of glutamate receptors and glutamate excitotoxicity during withdrawal period (that causes the breakdown of cytosolic calcium balance and oxidative stress). Thus, this aspect of the experimental design can be translated into an alcoholic patient hospitalized after a stroke.

Comparing our results with the previous data, 24 hours after the end of CAI, higher increase of nitrite levels (70%), but similar increase in LPO (80%), was observed in cortex ([9] and this work). Similar levels of LPO after eight days without ethanol intake, despite the decreased nitrite concentrations, suggest that, in addition to nitric oxide, other reactive species and mechanisms common for both alcoholism and abstinence (increased O_2_^•^ and OH^•^, NOX, phospholipase induction, etc.) could be playing an essential role in the deleterious consequences of oxidative stress [23].

The current findings demonstrate for the first time that CAI induces a deleterious synergism effect with stroke in behavioral and biochemical parameters.

Ischemia decreased cellular density and exacerbated neurodegeneration (loss of 88% of Neu-N-positive cells) in the cerebral cortex. These results are in accordance with previous data showing high neurodegeneration after seven days of the tissue infarction [19]. CAI, followed by eight days of abstinence, also reduced Neu-N-positive cells in 40%. The same immunostaining in the motor cortex, performed 24 h after the end of CAI, revealed about 40% of neuronal loss [9]. Therefore, we can hypothesize that the neurodegeneration observed in the present study is mainly due to CAI protocol with a minor role for the eight days of abstinence. The association of CAI and ischemia increased the loss of neuronal cell bodies, supporting the hypothesis that the synergy observed in both behavioral and oxidative stress analyses underlies an important neurodegenerative component.

Several mechanisms, including glial activation and neuroinflammation, have been proposed to explain the neurodegeneration induced by CAI and ischemia. Glial activation is a key element observed in animals treated with ethanol for 25 days (5 g/kg followed by 3 g/kg every 8 h, alternating 4 days of intoxication with 3 days of abstinence) [27]. However, it is not still clear if this activation is the cause or the consequence of the ethanol-induced neurotoxicity. Inflammatory events during CAI are associated with the activation of Toll-like receptors (TLR2, TLR3, and TLR4) by ethanol, with the consequent nuclear transcription factor kappa B (NF-*κ*B) induction and the increased expression of inflammatory mediators (COX-2, iNOS, TNF-*α*, IL-1*β*, and IL-6, among others) [24, 28].

Previously, the reduced microglial density was observed eight days after CAI [5]. Still, the possible withdrawal syndrome does not seem to play a major role in such effect because a similar decrease of microglial activation was detected (using IBA1-positive cells) 24 h after the last ethanol administration [9]. Also, a previous study already demonstrated that CAI could increase the levels of inflammatory mediators (iNOS, COX-2, and IL-1*β*) in the cerebral cortex and astrocytes culture, with the absence of microglial activation [29]. In accordance with this view, a significant astrogliosis was observed eight days after the end of CAI. However, in this case, alcohol withdrawal period may have influenced this response since astrocytic population was significantly reduced 24 h after the end of CAI [9]. According to this hypothesis, a previous study demonstrated a significant decrease in the number of astrocytes in rats exposed to CAI for 60 days and the occurrence of astrogliosis after three days of abstinence [30].

Stroke significantly increased astrocytic populations. Ischemic damage has been already characterized by the rapid recruitment and activation of astrocytes and microglial cells towards the injury core [31], probably triggered by TLR activation, proinflammatory mediators, and chemotactic factors.

Surprisingly, the association of CAI and ischemia induced significant lower levels of astrocytic activation than those caused by ischemia alone. These findings suggest that ischemia-induced astrogliosis may be limited by ethanol-induced cellular degeneration before the stroke [9].

The increased proliferation and reactivity of astrocytes play an important role in limiting the damage associated with focal ischemia. Spontaneous recovery after ischemia is directly associated with the formation of an astroglial scar (accumulation of these cells in the perinuclear region), which prevents its expansion [32]. A reduced astrocytic population could therefore influence the progression of the ischemic process by delaying or attenuating the formation of the glial scar. This would also affect the neurodegeneration in the penumbra region and the behavioral outcome.

Moreover, CAI affected not only the stroke outcome, but also the response to the therapy with minocycline. Previous studies demonstrated that minocycline is able to cross the blood brain barrier, promoting neuroprotective effects on the central nervous system after i.p. administration [11, 33, 34]. For each insult (CAI or stroke), protective effects of minocycline were more evident in stroke than in CAI (data not shown), perhaps because only 7 days of treatment occurred during the withdrawal syndrome, after 55 days of CAI. In regard to stroke, treatment with minocycline was effective in preventing/reversing deleterious alterations in behavioral and oxidative parameters. Minocycline treatment alone did not alter any behavioral parameter (data not shown) but it displayed a significant antioxidant effect in the motor cortex (reduced MDA and nitrite levels when compared to those of the control). The mechanisms underlying this latter effect may include decrease of ROS release by the cell (an indirect effect of the drug) and/or the direct scavenging of species such as peroxynitrites (minocycline is able to display an efficacy equivalent to alpha-tocopherol) [35]. The reduced tendency of deleterious side effects with minocycline treatment, in addition to the results over stroke and comorbidity, gives support to the therapeutic use of this drug.

In the association of CAI and stroke, minocycline efficacy was also evident for horizontal ambulation (crossed squares) and motor coordination (falls in rotarod). However, only partial efficacy for the latency to start movement, vertical exploration (rearing), and oxidative stress (MDA and nitrite levels) was observed. This suggests that an increased dose of minocycline, longer periods of treatment with this drug, or the additional use of an antioxidant in the therapeutic protocol must be considered for an adequate treatment of the comorbidity.

Despite the intense neuronal death (more than 75%) and the decrease in total number of cells in the motor cortex caused by ischemia, seven days of minocycline treatment was sufficient to normalize the latter parameter and partially prevent neuronal death. Although neuronal death was even more evident in the comorbidity, minocycline still elicited a significant protective effect. Since neurons are very sensitive to deleterious consequences of oxidative stress, the cotreatment with an antioxidant (as proposed above) may enhance the minocycline protective effect on neuronal tissue in comorbidity cases. Interestingly, treatment with minocycline also prevented astrocytic activation and partially protected against microglial activation caused by association of CAI and stroke.

The mechanisms underlying the benefits of minocycline remain unclear. The first and most widely studied pharmacological effect of minocycline has been its ability to inhibit glial activation [36]. This action is directly and selectively focused on microglial activation associated with neuroinflammation development (named type M1, and different than microglial activation type M2, associated with neuroprotection). M1 activation increases activity of NF*κ*B, with a consequent increased expression of TNF-*α*, interleukin-1*β*, IFN-*γ*, COX-2/prostaglandins, and NOS. By inhibiting this pathway, minocycline reduces the expression of NF*κ*B and the production of inflammatory mediators [37].

Also, minocycline decreases lesion area and neuronal loss by inhibiting matrix metalloproteinase 9 (MMP-9) activity, a zinc-dependent endopeptidase released by neurons [38]. By cleaving structural compounds of the matrix, this enzyme contributes to worsening the excitotoxicity process, neuronal damage, and cell death, affecting the blood brain barrier integrity.

## 5. Conclusion

The present study reinforces and extends previous evidence that CAI can exacerbate the deleterious effects of stroke, not only in behavior outcomes but also in altering cellular (worsening neuronal death and astrocytic activation) and biochemical responses (with a synergic deleterious effect in oxidative stress). Moreover, CAI also reduces the benefits of minocycline treatment against the motor impairments, oxidative stress, and neuronal loss induced by ischemia, possibly requiring adjustments in therapy.
